# Internet and Mobile Phone Addiction in Individuals Aged 15-50 Years

**DOI:** 10.1192/j.eurpsy.2025.963

**Published:** 2025-08-26

**Authors:** E. Szreder, K. Krysta

**Affiliations:** 1 Clinical Psychiatric Hospital SPZOZ in Rybnik, Rybnik; 2 Department of Rehabilitation Psychiatry, Medical University of Silesia, Katowice, Poland

## Abstract

**Introduction:**

Mobile phone addiction is a behavioral disorder characterized by the inability to function in daily life without a smartphone, most often with unlimited access to the internet. “Phone addicts” perceive their mobile phone as the most important tool for daily contact with others.

**Objectives:**

The aim of our study was to examine internet and mobile phone addiction in individuals aged 15-50 years.

**Methods:**

The study involved 107 participants who regularly use mobile phones and access the internet. The participants completed an online survey as part of the study. The Kimberly Young test was used to assess internet addiction. The test consisted of 7 questions with “yes” or “no” responses. A score of ≥4 points was considered indicative of addiction. To assess mobile phone addiction, a test consisting of 10 questions was used. The questions concerned attitudes towards SMS messages, phone calls, mobile network operator promotions, and mobile phone use. Each question had 3 possible answers. Providing answers marked with point “c” to 5 or more questions was considered indicative of mobile phone addiction. The only inclusion criteria were: being over 14 years old and owning a mobile phone with internet access.

**Results:**

The study involved 71 women, accounting for 66.4% of the respondents, and 36 men, accounting for 33.6% of the respondents. The age of the participants ranged from 16 to 50 years, with the average age being approximately 22.8 years. Among the study participants, 35.5% had higher education, 43.9% had secondary education, and 20.6% had primary education. 54.2% of the respondents lived in urban areas, while 45.8% lived in rural areas.

The questionnaire included a question regarding the amount of time spent online each day. According to the respondents’ answers, the average time spent online was approximately 358 minutes. The next question concerned the time spent on phone calls or text messages. The average time spent on this was approximately 75 minutes.

The results of the study indicated that 20 individuals, or 18.7% of the respondents, were addicted to the internet. Six individuals, or 5.6%, were addicted to their phones. Two respondents, or 1.9%, were addicted to both the internet and their phones. The data obtained were subjected to statistical analysis using Statistica software.

A statistically significant correlation was found between the degree of mobile phone addiction and the amount of time spent using the phone. The longer the time spent on the phone, the greater the degree of mobile phone addiction.

**Conclusions:**

Age significantly affects the degree of internet addiction. Among older individuals, the level of internet addiction is lower. The amount of time spent using the internet does not affect the degree of addiction. As the duration of mobile phone use increases, the intensity of mobile phone addiction also increases. Age does not affect the degree of mobile phone addiction.

**Disclosure of Interest:**

None Declared

